# Survival and complication analyses of avulsed and replanted permanent teeth

**DOI:** 10.1038/s41598-020-59843-1

**Published:** 2020-02-18

**Authors:** Daniel David Müller, Ricarda Bissinger, Marcel Reymus, Katharina Bücher, Reinhard Hickel, Jan Kühnisch

**Affiliations:** Department of Conservative Dentistry and Periodontology, School of Dentistry, Ludwig-Maximilians Universität, München, Munich Germany

**Keywords:** Dental diseases, Paediatric research

## Abstract

This retrospective clinical study investigated the survival probability of avulsed and replanted permanent teeth in relation to functional healing, replacement and inflammatory resorption. The explorative data analysis included data from 36 patients and 49 replanted permanent teeth with a minimum observation time of 60 days; the patients were generally treated according to the current guidelines of the International Association of Dental Traumatology at the university hospital in Munich, Germany, between 2004 and 2017. The mean observation period was 3.5 years. Functional healing was observed in 26.5% (N = 13/49) of the included avulsion cases. In comparison, replacement resorption affected 51.0% (N = 25/49) of the replanted teeth, of which 24.0% (N = 6/25) were lost over the course of years (mean, 6.1 years). In contrast, inflammatory resorption resulted in the early loss of all replanted teeth (mean, 1.7 years) and affected 22.5% (N = 11/49) of all the monitored teeth. Therefore, it can be concluded that tooth avulsion remains a severe dental injury with an unpredictable prognosis. This topic demands further fundamental research aiming to maintain and/or regenerate the periodontal ligament after tooth avulsion, particularly in association with non-physiological tooth rescue.

## Introduction

**Figure 1:**
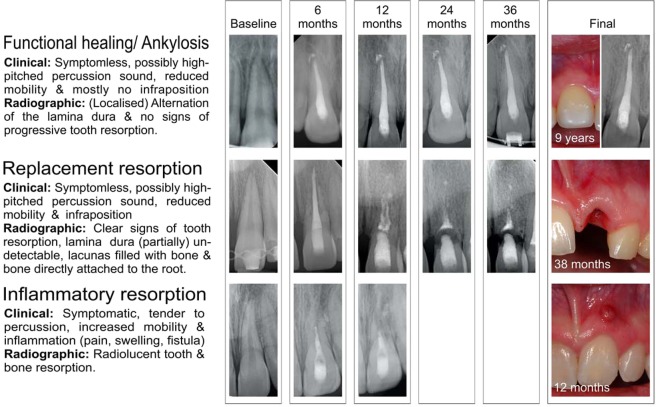
Overview of potential diagnoses for avulsed permanent teeth after replantation.

Tooth avulsion is defined as the complete loss of a tooth out of the alveolar bone socket as a result of an accident and represents a severe traumatic dental injury (TDI). Tooth avulsion mostly affects incisors in children and adolescents and is often associated with an unpredictable outcome and long-term treatment burden^1–3^. After replantation of the tooth, the prognosis commonly remains uncertain. Replacement resorption or inflammatory resorption are probable adverse outcomes in comparison to the more favourable functional healing (Fig. 1)^4^. Tooth resorption appears to be most likely in cases when connective tissue between the root cementum surface and the alveolar bone – the periodontal ligament (PDL) – is severely damaged. By mechanical destruction either during or after TDI, the cells on the root surface can be considerably impaired during the extra-oral dry time of the avulsed tooth. Therefore, immediate rescue of the avulsed tooth, without inducing further mechanical stress or contamination, by placement in a tooth rescue box with a physiological storage medium before the PDL cells dry out is of the utmost importance^5–8^. Physiologically, the roots of undamaged teeth are protected against resorption by a tissue layer of unmineralized organic cementum. Depending on the severity of the traumatic defect, the number of surviving PDL cells and, consequently, the cementum’s ability to regenerate, this layer may no longer act as a sufficient barrier between the dental hard tissue and the adjacent bone, thus no longer protecting the root from active clastic cells. Consequently, the PDL and the root structure might be involved in (physiological) bone remodelling processes and be replaced partially or completely by bone. In favourable cases, this effect will appear in only smaller demarcated areas for a limited period, leading to an acceptable prognosis, even if ankylosis and/or infraposition occur (“functional healing”). In more severe cases, resorption may continue, resulting in a complete loss of the root structure. If this final state is reached, incidental loss of the remaining tooth crown is to be expected (“replacement resorption”). In addition, pathological stimuli, such as bacterial contamination or infection and the associated toxins, necrotic cell debris, and mechanical stress, may enhance the resorption process and lead to rapid tooth loss in most cases (“inflammatory resorption”)^9–12^. These cellular processes are influenced by several other factors, such as the individual’s medical and dental characteristics and the quality of on-site primary (emergency) and sequential dental treatment, and therefore affect the overall prognosis of tooth survival.

The best practice recommendations include storage, replantation and splinting of the tooth. While these guidelines are constantly revised by experts^13–15^, data on the clinical efficacy of these treatment recommendations are sparse^16–20^. Therefore, this retrospectively designed clinical study aimed to analyse the prognosis for avulsed and replanted permanent teeth regarding longevity and complications in relation to relevant influential variables, e.g., dry storage time, extra-alveolar storage time, storage media, splinting time, and time to initiation of root canal treatment.

## Materials and Methods

### Ethical approval

All the procedures in these studies involving human participants were performed in accordance with the ethical standards of the institutional research committee and with the 1964 Helsinki Declaration and its later amendments or comparable ethical standards. The present study protocol was approved by the ethics committee of the Medical Faculty at the Ludwig-Maximilians University (LMU) of Munich (No. 670-16). Informed consent was obtained from each participant included in the study and/or their legal guardians.

### Standardized review of patient records

The patient documentation system at the Department of Conservative Dentistry and Periodontology at the LMU of Munich, Germany (university hospital), which includes paper patient files and, from 2012 onwards, files stored in a digital documentation system (Highdent Plus, CompuGroup Medical Dentalsysteme GmbH, Koblenz, Germany, version 5.57), was systematically screened by two dental professionals (DDM, RB). The records of patients who came to the university hospital with the request for examination and/or treatment of at least one avulsed permanent tooth from January 2004 to June 2017 were selected. Corresponding follow-up data were collected until June 2018. Teeth that were lost due to non-traumatic causes, such as caries, periodontal diseases, developmental defects or other diagnoses, were not considered. To enable structured data acquisition, an electronic case report form (EpiData, EpiData Association, Odense, Denmark, version V4.0.2.49) was designed, and all relevant information was entered. This included the individual patient information, the cause of the accident, on-site primary care (tooth rescue procedure, time periods, storage medium) and prior dental treatment to the extent to which this information could be obtained from the patient’s description, as well as details about the diagnosis, treatment and monitoring at the university hospital. If available, dental and medical reports, radiographs and photographs were used as an additional source to complement the dataset. Undocumented details were considered “missing information”.

### Study population

Initially, 32 male and 29 female patients (mean age, 13.8 years; range, 5–82 years) were identified, with 80 avulsed permanent teeth (74 upper incisors, 3 lower incisors, 2 upper canines, 1 lower canine) among them. Among 14 patients, 20 teeth were not replanted (teeth not recovered or too damaged: N = 10; severe medical condition: N = 6; other reasons: N = 4). To provide reliable conclusions about the course of treatment, the minimum observation time for this study was set to 60 days. This resulted in the exclusion of 11 patients (11 teeth) who were not seen for subsequent treatment and/or recall at the university hospital. Consequently, the final study population consisted of 36 patients (20 males and 16 females) with 49 replanted avulsed permanent teeth (1.36 replanted teeth per individual) (45 upper incisors, 2 lower incisors, 2 upper canines). This group comprised 24 patients with 32 teeth who underwent primary trauma management at the university hospital and 12 patients with 17 teeth who received dental care by an external dental health care provider (*alio loco*) and were seen and/or treated at the university hospital after.

### Diagnostic and treatment principles

The International Association of Dental Traumatology (IADT) offers guidelines for the management of TDI, including the treatment of avulsed permanent teeth^15^. The treatment principles at the university hospital followed the current best practice recommendations and some of the recommendations for treatment options based on consensus opinions. Patients seeking trauma care at the university hospital were immediately treated according to an emergency protocol. Even prior to obtaining the medical and accident-related history, all the avulsed teeth were instantly placed in a tooth rescue box (SOS Zahnrettungsbox, Miradent, Hager & Werken GmbH & Co. KG, Duisburg, Germany; Dentosafe, Medice, Iserlohn, Germany) by the first responding dental professional if these teeth were not already stored in a physiological medium or replanted before presentation. A tooth rescue box contains a physiological medium that preserves the vitality and proliferative capacity of PDL cells of isolated teeth. This physiological medium is pH-balanced, contains inorganic salts, amino acids and further ingredients such as glucose, buffering agents, vitamins and a preservative^21^. The emergency protocol included an initial medical examination to exclude central neurological damage and other severe bodily injuries, collection of the full medical and specific trauma history, instructions for tetanus protection, clinical and radiographic diagnostics, and treatment planning, aiming for replantation of the avulsed teeth as soon and as accurately as possible. In addition, the patients and/or guardians were educated on the treatment options, risks, follow-up procedures and possible outcomes as well as the related preparations and the actual replantation process. The avulsed permanent teeth were replanted according to the following procedure after the administration of local anaesthesia: The root surface and alveolar socket were examined and cleaned with saline. If required, repositioning and adaptation of the socket and adjacent tissues was performed. Then, individualisation and fixation of a semi-rigid splint (titanium trauma splint, TTS, Medartis AG, Basel, Switzerland) to adjacent teeth was performed prior to replantation of the affected teeth. Subsequently, the emergency procedure was completed by careful replantation, manual repositioning with slight digital pressure and fixation of the avulsed teeth to the splint with a flowable composite, followed by clinical and radiographic verification of adequate positioning. Suturing of the soft tissue was provided, if necessary. Antibiotics were prescribed on an individual basis. No extra-oral root canal treatment was performed, as the primary goal was the fastest possible and most gentle replantation in order to preserve PDL cell vitality.

All the patients were offered to be admitted according to the follow-up procedure of the university hospital for further dental treatment and long-term evaluation of the replanted teeth, including radiographic monitoring after 3, 6 and 12 months followed by a yearly recall thereafter. In recall sessions at the university hospital, the teeth and their surroundings were inspected visually as well as manually, and their mobility, percussion and sensitivity were tested using a refrigerant spray (Kältespray, Orbis, Münster, Germany) and/or electric pulp test (EPT, Parkell, Inc., Farmingdale, NY, USA), following a standardized examination protocol.

In all mature teeth with closed apices, preparation of an access cavity to the pulp chamber (trepanation) was executed, followed by intracanal medication via an aqueous calcium hydroxide solution (Ultracal, Ultradent Products GmbH, Köln, Germany) in a timely manner after replantation, depending on the sensitivity testing result, patient compliance and time and place of primary dental care^22^. In replanted teeth with immature root development, apexification^23^ was performed when consistent negative sensitivity testing for approximately 3 months indicated that no spontaneous revascularization of the traumatized pulp^24^ had occurred. Generally, the trauma splint was removed after endodontic treatment had been initiated. A definitive root canal filling with gutta-percha was performed in a timely manner, following trepanation, until 2010. Thereafter, a minimum calcium hydroxide application time of 12 months was considered appropriate to ensure the most effective decontamination of the root canal system^22,25^ and to rule out any rapid resorption processes requiring an early removal of applied definitive root canal fillings. Treatment protocols for avulsed teeth primarily treated by an external dental care provider may have differed.

### Statistics

**Figure 2:**
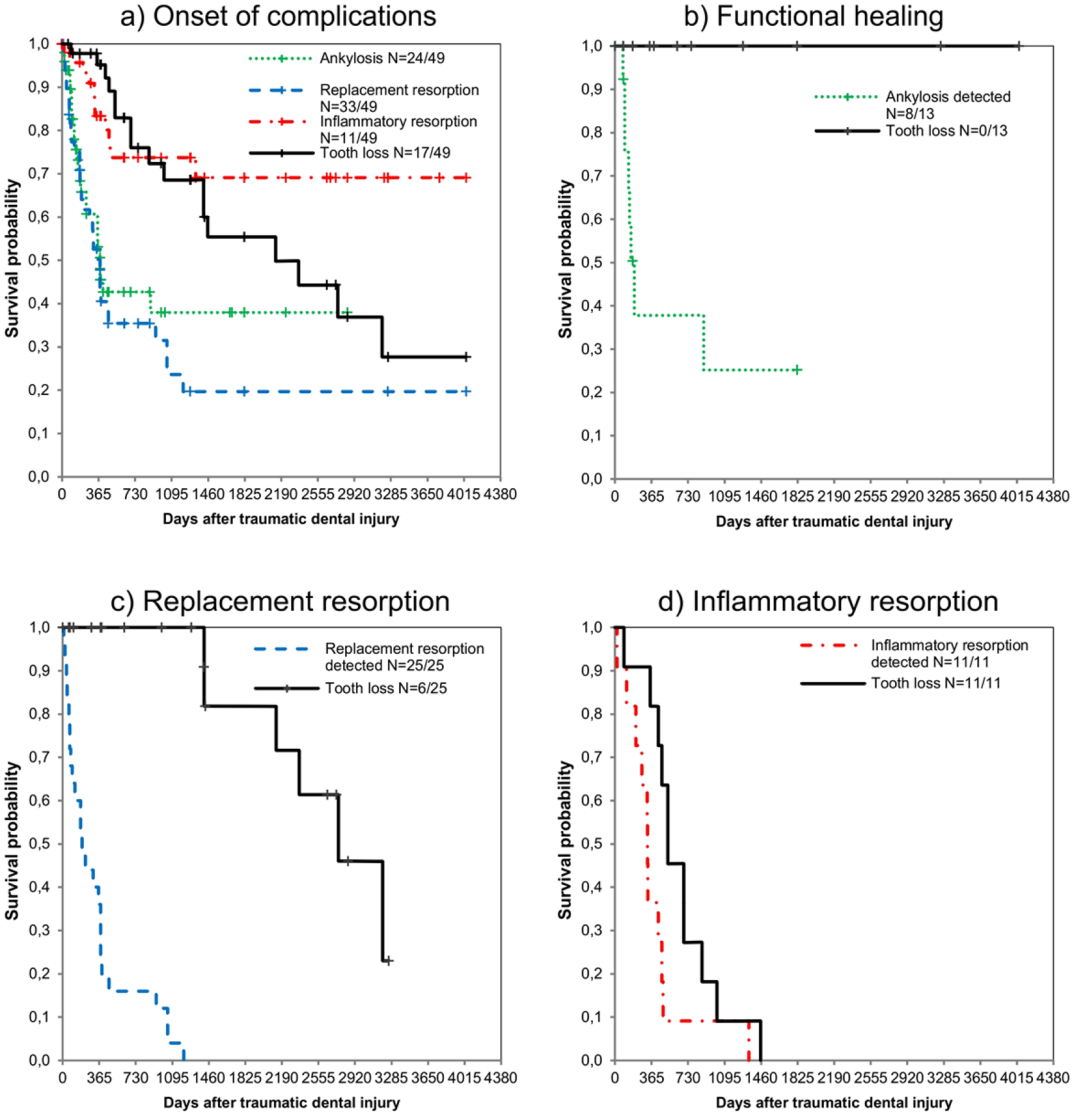
The Kaplan-Meier estimated survival curves for avulsed and replanted teeth with the onset of potential complications, including tooth loss. (**a**) All documented complications in all observed teeth (N = 49), irrespective of the final diagnosis; a single tooth might be linked to multiple diagnoses at different times. (**b**) Cases with a final diagnosis of functional healing/ankylosis (N = 13); no teeth were lost in this category. (**c**) Cases with a final diagnosis of replacement resorption (N = 25). (**d**) Cases with a final diagnosis of inflammatory resorption (N = 11).

All data were exported from the compiled EpiData database into an Excel (Office 365 Excel, Microsoft Corporation, Redmond, WA, USA, version 1804) sheet in a CSV file for further analysis. The descriptive data analysis was performed in Excel and SPSS (SPSS Statistics for Windows, IBM Company, Armonk, NY, USA, version 21.0.1). Analysis of the lifetime data included Kaplan-Meier estimations^26^. The survival curves show a summary of the survival experience of avulsed and replanted teeth, first for the entire study population that experienced complications, including tooth loss, in relation to the time elapsed since the accident. In this illustration, a single tooth might be linked to multiple complications at different time points (Fig. 2a). Second, the final diagnosis of each replanted tooth was utilized to assign each tooth to the corresponding category of functional healing/ankylosis, replacement resorption or inflammatory resorption (Fig. 2b–d). The estimated survival curves present the onset of the respective complication separately from tooth loss.

## Results

A total of 36 patients with a mean of 1.36 avulsions per individual were included. Accidents occurred during outdoor activities (N = 7 patients, 19.4%), at school (N = 7, 19.4%), in public places and on public transportation (N = 6, 16.7%), at home (N = 5, 13.9%), or at an unspecified location (N = 11, 30.6%). Most of the avulsions of this study population occurred due to falls (N = 16, 44.4%), while injuries inflicted by violence (N = 3, 8.3%), traffic accidents (N = 2, 5.6%), syncope (N = 2, 5.6%) and sports (N = 1, 2.8%) seemed to play only a minor role. The cause of the accident was not identifiable in 12 cases (33.3%).

The mean observation period for all the included 49 avulsed and replanted teeth amounted to 3.5 years, with a standard deviation (SD) of 3.2 years, ranging from 0.2 years to 11.1 years. Approximately two-thirds (N = 32, 65.3%) of all teeth were treated primarily at the university hospital, mostly on the day of the accident. The remaining one-third of all cases (N = 17, 34.7%) were referred to the university hospital during the monitoring process within a mean of 210 days (SD, 455.8 days; range, 0.0–1814 days) after the TDI. According to the patient recordings, the avulsed teeth remained in a non-physiological environment until storage in a physiological medium for a mean of 82 min (SD, 76.9 min; range, 1–300 min) and were replanted after an average time period of 173 min (SD, 203 min; range, 5–1110 min) after the initial accident. No spontaneous pulp revascularization could be observed. Removal of the definitive root canal fillings was indicated in 7 (36.8%) out of 19 teeth due to ongoing inflammatory (N = 3, 42.9%) or replacement resorptions (N = 4, 57.1%). The details regarding the time intervals in relation to the dental measures and the setting of primary dental care can be found in Table 1.

**Table 1:** Distribution of time intervals for crucial parameters determined for replanted permanent teeth, depending on the location of primary trauma treatment (N = 49).

Time interval	Setting of primary care after dental trauma	Mean	SD	Min	Max
Observation period [d]	University hospital (N = 32)	1416.4	1269.3	62	4037
	External dental care provider (N = 17)	973.0	885.1	72	3220
TDI to first visit at university hospital [d]	University hospital (N = 32)	0.1	0.2	0	1
	External dental care provider (N = 17)	210.0	455.8	0	1814
TDI to storage of tooth in medium: Dry storage [min]	University hospital (N = 13)	93.1	79.3	5	300
	External dental care provider (N = 8)	63.3	69.0	1	230
	Missing information (N = 28)	—	—	—	—
TDI to replantation: Extra-alveolar storage [min]	University hospital (N = 21)	205.8	233.2	60	1110
	External dental care provider (N = 10)	105.0	78.1	5	250
	Missing information (N = 18)	—	—	—	—
Splinting time [d]	University hospital (N = 32)	24.5	14.3	8	65
	External dental care provider (N = 13)	62.3	74.4	10	219
	Missing information (N = 4)	—	—	—	—
TDI to trepanation [d]	University hospital (N = 32)	36.3	30.2	0	158
	External dental care provider (N = 12)	115.8	142.9	0	423
	Missing information (N = 5)	—	—	—	—
TDI to definitive root canal filling [d]	University hospital (N = 14)	152.4	214.4	8	886
	External dental care provider (N = 5)	214.4	194.0	0	444
	No root canal filling (N = 26)	—	—	—	—
	Missing information (N = 4)	—	—	—	—
Root canal filling to removal [d] (N = 19)	University hospital (N = 5)	616.6	473.7	214	1424
	External dental care provider (N = 2)	219.0	0.0	219	219
	No revision (N = 12)	—	—	—	—
TDI to loss of tooth [d] (N = 17)	University hospital (N = 9)	1257.8	835.1	89	2755
	External dental care provider (N = 8)	1133.6	941.6	351	3197

The loss of replanted teeth occurred in 17 cases (N = 17/49, 34.7%; inflammatory resorption: N = 11/17, 64.7%; replacement resorption: N = 6/17, 35.3%); thus, 32 teeth (N = 32/49, 65.3%) were preserved (replacement resorption: N = 18/32, 56.2%; functional healing: N = 14/32, 43.8%). Tooth loss occurred on average after 3.2 years (SD, 2.5 years; range, 0.2–8.8 years). With regard to the underlying causes, an average period of 1.7 years (SD, 1.0 year; range, 0.2–4.0 years) in the cases of inflammatory resorption and an average period of 6.1 years (SD, 1.8 years; range, 3.9–8.8 years) in the cases of replacement resorption were observed. More descriptive data in relation to the relevant clinical variables are given in Tables 2 and 3.

**Table 2:** Descriptive survival data of replanted avulsed teeth with a minimum follow-up of 60 days in relation to relevant clinical variables.

Variable	Group	Tooth survival		Tooth loss	
		N	%	N	%
Dry storage time	0–15 min (N = 5)	4	80.0	1	20.0
	16–60 min (N = 6)	4	66.7	2	33.3
	>60 min (N = 10)	7	70.0	3	30.0
	Missing information (N = 28)	17	60.7	11	39.3
Extra-alveolar storage time	0–15 min (N = 2)	2	100.0	—	—
	16–60 min (N = 3)	3	100.0	—	—
	61–120 min (N = 12)	8	66.7	4	33.3
	>120 min (N = 14)	8	57.1	6	42.9
	Missing information (N = 18)	11	61.1	7	38.9
Storage medium	Tooth rescue box (N = 13)	11	84.6	2	15.4
	Normal saline (N = 4)	3	75.0	1	25.0
	Milk (N = 8)	4	50.0	4	50.0
	Intraoral (N = 1)	1	100.0	—	—
	Tap water (N = 2)	—	—	2	100.0
	Dry storage (N = 2)	2	100.0	—	—
	Missing information (N = 19)	11	57.9	8	42.1
Systemic antibiosis	Tetracycline (N = 13)	8	61.5	5	38.5
	Penicillin (N = 10)	8	80.0	2	20.0
	Other antibiotic (N = 5)	4	80.0	1	20.0
	No antibiotic (N = 5)	5	100.0	—	—
	Missing information (N = 16)	7	43.8	9	56.3
Timespan: TDI to trepanation	0–14 d (N = 17)	11	64.7	6	35.3
	15–21 d (N = 9)	6	66.7	3	33.3
	22–60 d (N = 8)	4	50.0	4	50.0
	>60 d (N = 10)	8	80.0	2	20.0
	Missing information (N = 5)	3	60.0	2	40.0
Splinting time	1–20 d (N = 22)	15	68.2	7	31.8
	21–40 d (N = 15)	9	60.0	6	40.0
	>41 d (N = 8)	6	75.0	2	25.0
	Missing information (N = 4)	2	50.0	2	50.0
Total	(N = 49)	32	65.3	17	34.7

**Table 3:** Descriptive data on functional healing, replacement resorption and inflammatory resorption for replanted avulsed teeth with a minimum follow-up of 60 days in relation to relevant clinical variables.

Variable	Group	Functional healing		Replacement resorption		Inflammatory resorption	
		N	%	N	%	N	%
Dry storage time	0–15 min (N = 5)	3	60.0	2	40.0	—	—
	16–60 min (N = 6)	2	33.3	2	33.3	2	33.3
	>60 min (N = 10)	2	20.0	5	50.0	3	30.0
	Missing information (N = 28)	6	21.4	16	57.1	6	21.4
Extra- alveolar storage time	0–15 min (N = 2)	2	100.0	—	—	—	—
	16–60 min (N = 3)	2	66.7	1	33.3	—	—
	61–120 min (N = 12)	2	16.7	10	83.3	—	—
	>120 min (N = 14)	2	14.3	6	42.9	6	42.9
	Missing information (N = 18)	5	27.8	8	44.4	5	27.8
Storage medium	Tooth rescue box (N = 13)	7	53.8	4	30.8	2	15.4
	Normal saline (N = 4)	1	25.0	3	75.0	—	—
	Milk (N = 8)	0	0.0	5	62.5	3	37.5
	Intraoral (N = 1)	1	100.0	—	—	—	—
	Tap water (N = 2)	—	—	2	100.0	—	—
	Dry storage (N = 2)	—	—	2	100.0	—	—
	Missing information (N = 19)	4	21.1	9	47.4	6	31.6
Systemic antibiosis	Tetracycline (N = 13)	3	23.1	6	46.2	4	30.8
	Penicillin (N = 10)	4	40.0	5	50.0	1	10.0
	Other antibiotic (N = 5)	2	40.0	2	40.0	1	20.0
	No antibiotic (N = 5)	2	40.0	3	60.0	—	—
	Missing information (N = 16)	2	12.5	9	56.3	5	31.3
Timespan: TDI to trepanation	0–14 d (N = 17)	5	29.4	8	47.1	4	23.5
	15–21 d (N = 9)	2	22.2	4	44.4	3	33.3
	22–60 d (N = 8)	2	25.0	4	50.0	2	25.0
	>60 d (N = 10)	4	40.0	4	40.0	2	20.0
	Missing information (N = 5)	1	20.0	4	80.0	—	—
Splinting time	1–20 d (N = 22)	8	36.4	10	45.5	4	18.2
	21–40 d (N = 15)	3	20.0	8	53.3	4	26.7
	>41 d (N = 8)	2	25.0	4	50.0	2	25.0
	Missing information (N = 4)	—	—	3	75.0	1	25.0
Total	(N = 49)	13	26.5	25	51.0	11	22.5

The Kaplan-Meier estimated survival curves in Fig. 2a show the highest probability for the detection of complications in the first two years after TDI and a consistent risk for tooth loss over the entire observation period. On closer examination of the individual categories, it becomes apparent that there is a high probability of tooth survival for replanted teeth that healed without complication or ankylosis (Fig. 2b). For teeth with replacement resorption but no inflammation (Fig. 2c), tooth loss was a probable outcome but was more likely to occur many years after the detection of resorption. In contrast, inflammatory resorption was likely to cause rapid tooth loss (Fig. 2d).

The cohort comprised of 25 (51%) immature teeth with open apertures at apices (>1 mm in radiographs) and 24 (49%) with closed apertures at apices (<1 mm). In general, no relevant differences were detected between immature and mature avulsed and replanted teeth with regard to the occurrence of complications (for details see supplementary online information).

## Discussion

The present retrospective clinical study reports key variables that are closely linked to TDI and its dental management (Tables 1–3) and, more importantly, investigates the long-term outcome of 49 avulsed and replanted permanent teeth (Fig. 2). The first noticeable aspect is that the observed outcomes are very heterogeneous; they range from symptom-free healing to inflammation and rapid tooth loss. This study found an overall tooth survival rate of 65.3% within a mean observation period of 3.5 years for replanted avulsed teeth. This is considerably inferior to the success rates of most other treatment procedures in modern dentistry, including dental traumatology, but it is in line with other reports on replanted avulsed teeth, showing survival rates between 50.0% and 83.3%^16–18,20,27,28^ and emphasizing the severity of this specific type of TDI. The most desirable outcome after avulsion is functional healing, which was observed in 26.5% (N = 13/49) of all monitored cases and did not lead to the loss of a single tooth during the observation period (Fig. 2b). In contrast, all the teeth showing inflammatory resorption (N = 11/49, 22.5%) were lost within the first 4 years after the accident, with the highest probability of loss within the first two years post-trauma (Fig. 2d). In comparison, the teeth showing replacement resorption (N = 25/49, 51.0%) may remain clinically asymptomatic for many years, resulting in a lower probability of tooth loss in the first three years after trauma (Fig. 2c). Tooth loss related to replacement resorption (N = 6/24, 25.0%) may occur as a long-term consequence mostly due to crown fractures following extensive root resorption. It can be concluded that replanted avulsed teeth that do not show any signs of resorption within the first three years after the accident are likely to remain symptom-free, but for teeth affected by resorption, tooth loss, possibly years after the accident, is a likely outcome. As most cases of avulsion occur in juvenile patients^3,29^, it is of clinical importance to preserve the tooth and surrounding bone tissue to provide optimal, definitive, prosthodontic rehabilitation under the possibility of later treatment with dental implants.

In this study no conclusive association between the stage of root development and potential complications in avulsed and replanted teeth was registered (supplementary information). This finding may support the initially mentioned hypothesis that the PDL protects the tooth against active osteoclastic cells. Thus, the severity of PDL damage may have a greater influence on the occurrence of resorptions and/or tooth loss than the stage of root development. Nevertheless, this assumption needs verification in future and larger cohort studies.

It is common knowledge that the prompt rescue and replantation of an avulsed tooth will enhance the probability of achieving a favourable outcome after trauma^15^. Based on the data of the included cases, optimal tooth rescue is a rare and exceptional occurrence in real-life settings, which is illustrated by the documented average dry storage time of 82 min and mean of 173 min until replantation (Table 1). Only two teeth, in the same patient, were replanted immediately on the accident site, and only three more were placed in a tooth rescue box within 15 min after the accident. This supports the recommendation that tooth rescue, often provided by non-professionals, is of the utmost importance for the fate of avulsed teeth. The widespread and sustainable availability of special physiological storage media and, more importantly, knowledge of how to manage the replantation of avulsed teeth on-site need a major boost in awareness in terms of medical first aid guidance. Considering that such proposals have already been made in the past, the uncertain coverage of costs and the unpredictability of the location and timing of TDI complicate the establishment of a close and ubiquitous tooth rescue service^30^. Furthermore, deviations in the treatment intervals were noticed (Table 2), despite all efforts to adhere to international guidelines^15^. In particular, in some cases, the splinting time was exceeded, which also resulted in delayed endodontic treatment (Table 1). This might be partially because of the lack of patient compliance.

Even less encouraging for clinicians is the low long-term survival probability of avulsed and replanted teeth and limited treatment options to control complications at a later stage. One reason for these issues is the early and unpredictable appearance of inflammatory resorption. Even if endodontic treatment is provided in a timely manner, inflammation and/or resorption can lead to the rapid destruction of dental hard and/or bone tissue, possibly leading to gutta-percha being integrated into the bone tissue. Experiencing the possibility of early destructive resorption led to modification of the endodontic treatment procedure in the present study population. After ~2010, definitive endodontic treatment was no longer carried out earlier than one year after TDI and was carried out only under the condition that no resorption was diagnosed to avoid the challenging removal of definite root canal filling material from the partially resorbed tooth and replacing bone.

To treat avulsed teeth after unfavourable tooth rescue, most techniques, until now, have aimed at delaying the onset and/or limiting the extent of replacement resorption. Panzarini *et al*.^31^ reviewed strategies for treating the root surface or PDL prior to replantation with a variety of agents, e.g., antibiotics, enamel matrix proteins, acid etching solutions, fluoride, corticosteroids, or bisphosphonates, but concluded that none of the reviewed reports offered a treatment that could prevent or heal ankylosis or replacement resorption in teeth with severely damaged PDL cells. New approaches for research activities based on the underlying cell biology are urgently needed, with the aim of finding a way to restore or rebuild the PDL to prevent tooth loss in replanted teeth after unfavourable tooth rescue^32^.

This longitudinal, retrospective clinical study has strengths and limitations. Mainly, the study was based on a longitudinal recall over several years of caring for patients who received dental care due to avulsion between 2004 and 2017. Long-term and detailed data for TDI cases, especially the avulsion of permanent teeth, have been rare in the last decade^16,17,20^; therefore, current information is required to reflect the present state of clinical practice. Although a practice-based, retrospective study design is often considered less optimal, particularly due to the possibility of missing information (Tables 1–3), this design might still be the most feasible approach to study the outcome of replanted permanent teeth in humans under real-life circumstances. The use of a randomized, prospective study design would have been more appropriate to collect data systematically, but it is difficult to subject patients, most of whom are underage and in an acute emergency situation, to the necessary preliminary procedure. One limitation of this study was that several patients with replanted teeth decided not to receive follow-up treatment at the university hospital, which resulted in a smaller sample size. Furthermore, only those cases with a minimum observation time of 60 days were included, which not only further reduced the sample size but also helped to limit the heterogeneity of the study population. However, several patients who did not receive immediate dental care at the university hospital after TDI but had been referred to the university hospital at a later time due to associated complications were included. This may have led to an overrepresentation of undesirable events in this case series. The small sample size limits the possible use of regression analyses. Therefore, solely descriptive statistical data are provided (Tables 1–3), presenting an overview of the current findings instead of detailed explorative analyses. To include larger numbers of cases, the urgent need for a well-standardized, multi-centre clinical study or specialist network aiming to investigate possible outcome scenarios after tooth avulsion should be emphasized.

## Conclusions

One-third of replanted avulsed teeth were lost during the mean observation period of 3.5 years, and only one out of four replanted teeth showed functional healing. Teeth were lost earlier in cases linked to inflammatory resorption (mean, 1.7 years) than in cases linked to replacement resorption (mean, 6.1 years). Therefore, it can be concluded that tooth avulsion remains a severe dental injury with an unpredictable prognosis, which demands new fundamental research aiming to maintain and/or regenerate the PDL after tooth avulsion.

## Supplementary information

Supplementary information.
