# Supplementary material for: Survival and complication analyses of avulsed and replanted permanent teeth
Source: Sci Rep. 2020 Feb 18;10:2841. doi: 10.1038/s41598-020-59843-1 (PMC7028940; doi:10.1038/s41598-020-59843-1)
Supplement: Supplementary file 1 — Supplementary information. [file 41598_2020_59843_MOESM1_ESM.docx]

**Supplementary information**

**Survival and complication analyses of avulsed and replanted permanent teeth**

Daniel David Müller, Ricarda Bissinger, Marcel Reymus, Katharina Bücher, Reinhard Hickel, Jan Kühnisch

Department of Conservative Dentistry and Periodontology, School of Dentistry, Ludwig-Maximilians Universität, München, Munich, Germany

**Table S1** Descriptive data of survived replanted avulsed teeth with a minimum follow-up of 60 days in relation to stage of root development.

|  |  | **Tooth survival** | | | |
| --- | --- | --- | --- | --- | --- |
| **Variable** | **Group** | **Immature teeth**  **(Open Apex)** | | **Mature teeth**  **(Closed Apex)** | |
|  |  | N | % | N | % |
| **Dry storage time** | 0-15 min (N=4) | 3 | 75.0 | 1 | 25.0 |
|  | 16-60 min (N=4) | 3 | 75.0 | 1 | 25.0 |
|  | >60 min (N=7) | 3 | 42.9 | 4 | 57.1 |
|  | Missing information (N=17) | 7 | 41.2 | 10 | 58.8 |
| **Extra-alveolar storage time** | 0-15 min (N=2) | 2 | 100.0 | - | - |
|  | 16-60 min (N=3) | 2 | 66.7 | 1 | 33.3 |
|  | 61-120 min (N=8) | 5 | 62.5 | 3 | 37.5 |
|  | >120 min (N=8) | 3 | 37.5 | 5 | 62.5 |
|  | Missing information (N=11) | 4 | 36.4 | 7 | 63.6 |
| **Storage medium** | Tooth rescue box (N=11) | 3 | 27.3 | 8 | 72.7 |
|  | Normal saline (N=3) | 1 | 33.3 | 2 | 66.7 |
|  | Milk (N=4) | 3 | 75.0 | 1 | 25.0 |
|  | Intraoral (N=1) | 1 | 100.0 | - | - |
|  | Tap water (N=0) | - | - | - | - |
|  | Dry storage (N=2) | 2 | 100.0 | - | - |
|  | Missing information (N=11) | 6 | 54.5 | 5 | 45.5 |
| **Systemic antibiosis** | Tetracycline (N=8) | 1 | 12.5 | 7 | 87.5 |
|  | Penicillin (N=8) | 4 | 50.0 | 4 | 50.0 |
|  | Other antibiotic (N=4) | 4 | 100.0 | - | - |
|  | No antibiotic (N=5) | 3 | 60.0 | 2 | 40.0 |
|  | Missing information (N=7) | 4 | 57.1 | 3 | 42.9 |
| **Timespan: TDI to trepanation** | 0-14 d (N=11) | 4 | 36.4 | 7 | 63.6 |
|  | 15-21 d (N=6) | 1 | 16.7 | 5 | 83.3 |
|  | 22-60 d (N=4) | 1 | 25.0 | 3 | 75.0 |
|  | >60 d (N=8) | 7 | 87.5 | 1 | 12.5 |
|  | Missing information (N=3) | 3 | 100.0 | - | - |
| **Splinting time** | 1-20 d (N=15) | 7 | 46.7 | 8 | 53.3 |
|  | 21-40 d (N=9) | 3 | 33.3 | 6 | 66.7 |
|  | >41 d (N=6) | 4 | 66.7 | 2 | 33.3 |
|  | Missing information (N=2) | 2 | 100.0 | - | - |
| **Total** | (N=32) | 16 | 50.0 | 16 | 50.0 |

**Table S2** Descriptive data of replanted and subsequently lost avulsed teeth in relation to stage of root development after a minimum follow-up of 60 days.

|  |  | **Tooth loss** | | | |
| --- | --- | --- | --- | --- | --- |
| **Variable** | **Group** | **Immature teeth**  **(Open Apex)** | | **Mature teeth**  **(Closed Apex)** | |
|  |  | N | % | N | % |
| **Dry storage time** | 0-15 min (N=1) | 1 | 100.0% | - | - |
|  | 16-60 min (N=2) | - | - | 2 | 100.0% |
|  | >60 min (N=3) | 2 | 66.7 | 1 | 33.3 |
|  | Missing information (N=11) | 6 | 54.5 | 5 | 45.5 |
| **Extra-alveolar storage time** | 0-15 min (N=0) | - | - | - | - |
|  | 16-60 min (N=0) | - | - | - | - |
|  | 61-120 min (N=4) | 1 | 25.0 | 3 | 75.0 |
|  | >120 min (N=6) | 2 | 33.3 | 4 | 66.7 |
|  | Missing information (N=7) | 6 | 85.7 | 1 | 14.3 |
| **Storage medium** | Tooth rescue box (N=2) | 1 | 50.0 | 1 | 50.0 |
|  | Normal saline (N=1) | 1 | 100.0 | - | - |
|  | Milk (N=4) | 1 | 25.0 | 3 | 75.0 |
|  | Intraoral (N=0) | - | - | - | - |
|  | Tap water (N=2) | - | - | 2 | 100.0 |
|  | Dry storage (N=0) | - | - | - | - |
|  | Missing information (N=8) | 6 | 75.0 | 2 | 25.0 |
| **Systemic antibiosis** | Tetracycline (N=5) | 1 | 20.0 | 4 | 80.0 |
|  | Penicillin (N=2) | 2 | 100.0 | - | - |
|  | Other antibiotic (N=1) | - | - | 1 | 100.0 |
|  | No antibiotic (N=0) | - | - | - | - |
|  | Missing information (N=9) | 6 | 66.7 | 3 | 33.3 |
| **Timespan: TDI to trepanation** | 0-14 d (N=6) | 3 | 50.0 | 3 | 50.0 |
|  | 15-21 d (N=3) | 1 | 33.3 | 2 | 66.7 |
|  | 22-60 d (N=4) | 1 | 25.0 | 3 | 75.0 |
|  | >60 d (N=2) | 2 | 100.0 | - | - |
|  | Missing information (N=2) | 2 | 100.0 | - | - |
| **Splinting time** | 1-20 d (N=7) | 3 | 42.9 | 4 | 57.1 |
|  | 21-40 d (N=6) | 2 | 33.3 | 4 | 66.7 |
|  | >41 d (N=2) | 2 | 100.0 | - | - |
|  | Missing information (N=2) | 2 | 100.0 | - | - |
| **Total** | (N=17) | 9 | 52.9 | 8 | 47.1 |

**Table S3** Descriptive data on replanted avulsed teeth showing functional healing after a minimum follow-up of 60 days in relation to the maturity of corresponding teeth.

|  |  | **Functional healing** | | | |
| --- | --- | --- | --- | --- | --- |
| **Variable** | **Group** | **Immature teeth**  **(Open Apex)** | | **Mature teeth**  **(Closed Apex)** | |
|  |  | N | % | N | % |
| **Dry storage time** | 0-15 min (N=3) | 2 | 66.7 | 1 | 33.3 |
|  | 16-60 min (N=2) | 1 | 50.0 | 1 | 50.0 |
|  | >60 min (N=2) | 1 | 50.0 | 1 | 50.0 |
|  | Missing information (N=6) | 1 | 16.7 | 5 | 83.3 |
| **Extra- alveolar storage time** | 0-15 min (N=2) | 2 | 100.0 | - | - |
|  | 16-60 min (N=2) | 1 | 50.0 | 1 | 50.0 |
|  | 61-120 min (N=2) | - | - | 2 | 100.0 |
|  | >120 min (N=2) | 1 | 50.0 | 1 | 50.0 |
|  | Missing information (N=5) | 1 | 20.0 | 4 | 80.0 |
| **Storage medium** | Tooth rescue box (N=7) | 2 | 28.6 | 5 | 71.4 |
|  | Normal saline (N=1) | - | - | 1 | 100.0 |
|  | Milk (N=0) | - | - | - | - |
|  | Intraoral (N=1) | 1 | 100.0 | - | - |
|  | Tap water (N=0) | - | - | - | - |
|  | Dry storage (N=0) | - | - | - | - |
|  | Missing information (N=4) | 2 | 50.0 | 2 | 50.0 |
| **Systemic antibiosis** | Tetracycline (N=3) | - | - | 3 | 100.0 |
|  | Penicillin (N=4) | 3 | 75.0 | 1 | 25.0 |
|  | Other antibiotic (N=2) | 2 | 100.0 | - | - |
|  | No antibiotic (N=2) | - | - | 2 | 100.0 |
|  | Missing information (N=2) | - | - | 2 | 100.0 |
| **Timespan: TDI to trepanation** | 0-14 d (N=4) | 1 | 25.0 | 3 | 75.0 |
|  | 15-21 d (N=2) | - | - | 2 | 100.0 |
|  | 22-60 d (N=2) | - | - | 2 | 100.0 |
|  | >60 d (N=4) | 3 | 75.0 | 1 | 25.0 |
|  | Missing information (N=4) | 1 | 100.0 | - | - |
| **Splinting time** | 1-20 d (N=8) | 3 | 37.5 | 5 | 62.5 |
|  | 21-40 d (N=3) | 1 | 33.3 | 2 | 66.7 |
|  | >41 d (N=2) | 1 | 50.0 | 1 | 50.0 |
|  | Missing information (N=0) | - | - | - | - |
| **Total** | (N=13) | 5 | 38.5 | 8 | 61.5 |

**Table S4** Descriptive data on replanted avulsed teeth showing replacement resorption after a minimum follow-up of 60 days in relation to the maturity of corresponding teeth.

|  |  | **Replacement resorption** | | | |
| --- | --- | --- | --- | --- | --- |
| **Variable** | **Group** | **Immature teeth**  **(Open Apex)** | | **Mature teeth**  **(Closed Apex)** | |
|  |  | N | % | N | % |
| **Dry storage time** | 0-15 min (N=2) | 2 | 100.0 | - | - |
|  | 16-60 min (N=2) | 2 | 100.0 | - | - |
|  | >60 min (N=5) | 2 | 40.0 | 3 | 60.0 |
|  | Missing information (N=16) | 8 | 50.0 | 8 | 50.0 |
| **Extra- alveolar storage time** | 0-15 min (N=0) | - | - | - | - |
|  | 16-60 min (N=1) | 1 | 100.0 | - | - |
|  | 61-120 min (N=10) | 6 | 60.0 | 4 | 40.0 |
|  | >120 min (N=6) | 2 | 33.3 | 4 | 66.7 |
|  | Missing information (N=8) | 5 | 62.5 | 3 | 37.5 |
| **Storage medium** | Tooth rescue box (N=4) | 1 | 25.0 | 3 | 75.0 |
|  | Normal saline (N=3) | 2 | 66.7 | 1 | 33.3 |
|  | Milk (N=5) | 3 | 60.0 | 2 | 40.0 |
|  | Intraoral (N=0) | - | - | - | - |
|  | Tap water (N=2) | - | - | 2 | 100.0 |
|  | Dry storage (N=2) | 2 | 100.0 | - | - |
|  | Missing information (N=9) | 6 | 66.7 | 3 | 33.3 |
| **Systemic antibiosis** | Tetracycline (N=6) | 1 | 16.7 | 5 | 83.3 |
|  | Penicillin (N=5) | 2 | 40.0 | 3 | 60.0 |
|  | Other antibiotic (N=2) | 2 | 100.0 | - | - |
|  | No antibiotic (N=3) | 3 | 100.0 | - | - |
|  | Missing information (N=9) | 6 | 66.7 | 3 | 33.3 |
| **Timespan: TDI to trepanation** | 0-14 d (N=9) | 4 | 44.4 | 5 | 55.6 |
|  | 15-21 d (N=4) | 1 | 25.0 | 3 | 75.0 |
|  | 22-60 d (N=4) | 1 | 25.0 | 3 | 75.0 |
|  | >60 d (N=4) | 4 | 100.0 | - | - |
|  | Missing information (N=4) | 4 | 100.0 | - | - |
| **Splinting time** | 1-20 d (N=10) | 6 | 60.0 | 4 | 40.0 |
|  | 21-40 d (N=8) | 2 | 25.0 | 6 | 75.0 |
|  | >41 d (N=4) | 3 | 75.0 | 1 | 25.0 |
|  | Missing information (N=3) | 3 | 100.0 | - | - |
| **Total** | (N=25) | 14 | 56.0 | 11 | 44.0 |

**Table S5** Descriptive data on replanted avulsed teeth showing inflammatory resorption after a minimum follow-up of 60 days in relation to the maturity of corresponding teeth.

|  |  | **Inflammatory resorption** | | | |
| --- | --- | --- | --- | --- | --- |
| **Variable** | **Group** | **Immature teeth**  **(Open Apex)** | | **Mature teeth**  **(Closed Apex)** | |
|  |  | N | % | N | % |
| **Dry storage time** | 0-15 min (N=0) | - | - | - | - |
|  | 16-60 min (N=2) | - | - | 2 | 100.0 |
|  | >60 min (N=3 | 2 | 66.7 | 1 | 33.3 |
|  | Missing information (N=6) | 4 | 66.7 | 2 | 33.3 |
| **Extra- alveolar storage time** | 0-15 min (N=0) | - | - | - | - |
|  | 16-60 min (N=0) | - | - | - | - |
|  | 61-120 min (N=0) | - | - | - | - |
|  | >120 min (N=6) | 2 | 33.3 | 4 | 66.7 |
|  | Missing information (N=5) | 4 | 80.0 | 1 | 20.0 |
| **Storage medium** | Tooth rescue box (N=2) | 1 | 50.0 | 1 | 50.0 |
|  | Normal saline (N=0) | - | - | - | - |
|  | Milk (N=3) | 1 | 33.3 | 2 | 66.7 |
|  | Intraoral (N=0) | - | - | - | - |
|  | Tap water (N=0) | - | - | - | - |
|  | Dry storage (N=0) | - | - | - | - |
|  | Missing information (N=6) | 4 | 66.7 | 2 | 33.3 |
| **Systemic antibiosis** | Tetracycline (N=4) | 1 | 25.0 | 3 | 75.0 |
|  | Penicillin (N=1) | 1 | 100.0 | - | - |
|  | Other antibiotic (N=1) | - | - | 1 | 100.0 |
|  | No antibiotic (N=0) | - | - | - | - |
|  | Missing information (N=5) | 4 | 80.0 | 1 | 20.0 |
| **Timespan: TDI to trepanation** | 0-14 d (N=4) | 2 | 50.0 | 2 | 50.0 |
|  | 15-21 d (N=3) | 1 | 33.3 | 2 | 66.7 |
|  | 22-60 d (N=2) | 1 | 50.0 | 1 | 50.0 |
|  | >60 d (N=2) | 2 | 100.0 | - | - |
|  | Missing information (N=0) | - | - | - | - |
| **Splinting time** | 1-20 d (N=4) | 1 | 25.0 | 3 | 75.0 |
|  | 21-40 d (N=4) | 2 | 50.0 | 2 | 50.0 |
|  | >41 d (N=2) | 2 | 100.0 | - | - |
|  | Missing information (N=1) | 1 | 100.0 | - | - |
| **Total** | (N=11) | 6 | 54.5 | 5 | 45.5 |

**Figure S1** The Kaplan-Meier estimated survival curves for *immature* avulsed and replanted teeth in relation to the onset of potential complications, including tooth loss. a) All documented complications in all observed teeth (N=25), irrespective of the final diagnosis; a single tooth might be linked to multiple diagnoses at different times. b) Cases with a final diagnosis of functional healing/ankylosis (N=5); no teeth were lost in this category. c) Cases with a final diagnosis of replacement resorption (N=14). d) Cases with a final diagnosis of inflammatory resorption (N=6).

1. Onset of complications b) Functional healing
2. Replacement resorption d) Inflammatory resorption

**Figure S2** The Kaplan-Meier estimated survival curves for *mature* avulsed and replanted teeth in relation to the onset of potential complications, including tooth loss. a) All documented complications in all observed teeth (N=24), irrespective of the final diagnosis; a single tooth might be linked to multiple diagnoses at different times. b) Cases with a final diagnosis of functional healing/ankylosis (N=8); no teeth were lost in this category. c) Cases with a final diagnosis of replacement resorption (N=11). d) Cases with a final diagnosis of inflammatory resorption (N=5).

1. Onset of complications b) Functional healing
2. Replacement resorption d) Inflammatory resorption
